# Supplementary figures and images for: Maternal lipid mobilization is essential for embryonic development in the malaria vector Anopheles gambiae
Source: PLoS Biol. 2024 Dec 17;22(12):e3002960. doi: 10.1371/journal.pbio.3002960 (PMC11703037; doi:10.1371/journal.pbio.3002960)

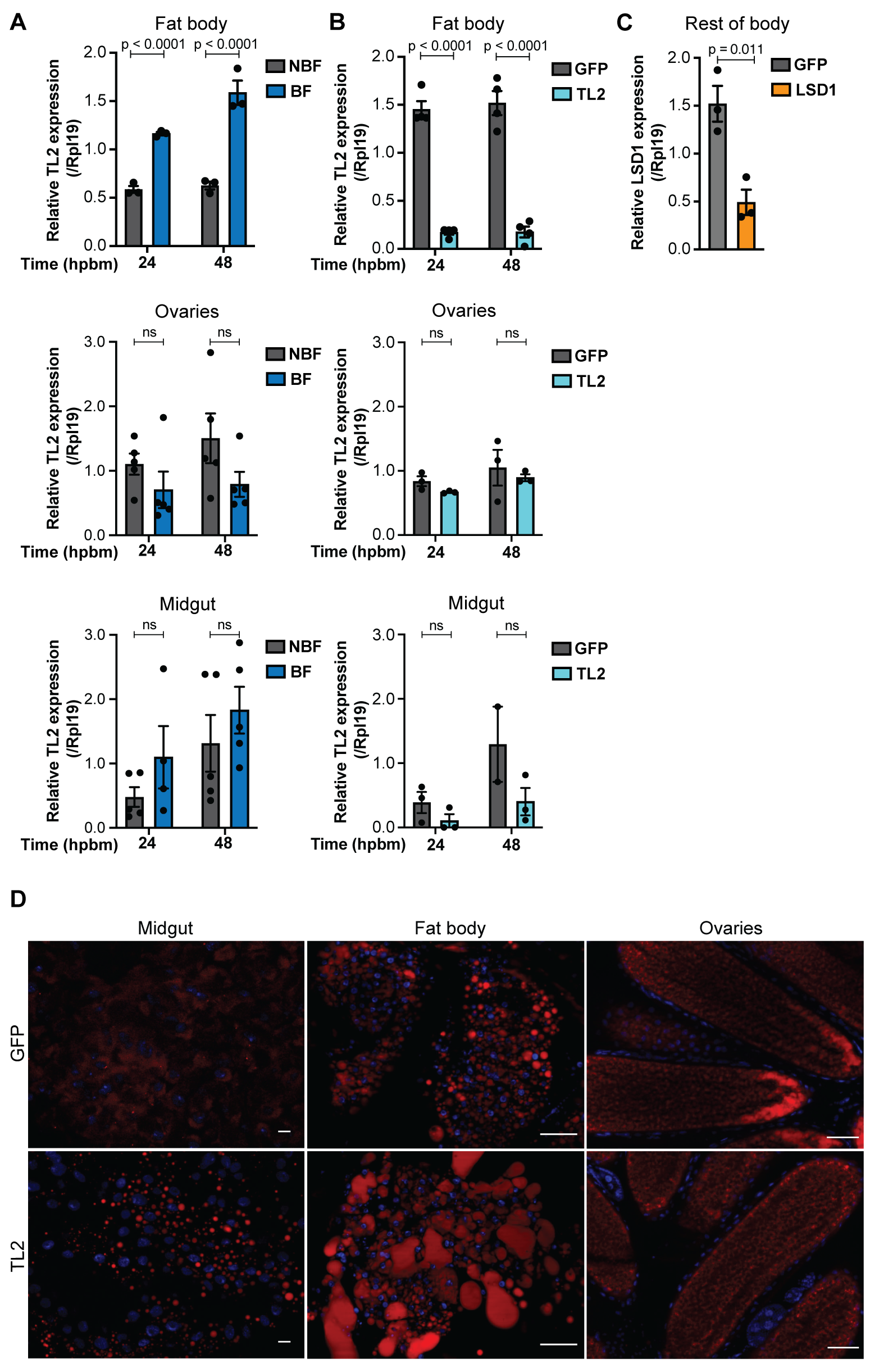

Supplement: S1 Fig — (A) AgTL2 is significantly induced in the fat body but not the ovaries or midgut of females at 2 time points after blood feeding (hPBM = hours post blood meal) compared to non-blood fed (NBF) controls. (Ordinary 2-way ANOVA, Šídák’s multiple comparisons correction) (B) AgTL2 levels after blood feeding are significantly reduced upon RNAi injections in the fat body but not the ovaries or midgut (Ordinary 2-way ANOVA, Šídák’s multiple comparisons correction). (C) AgLSD1 levels are reduced upon RNAi injections at 3 d post injection in whole body minus head samples (Rest of body) (Unpaired t test). Each dot in A–C represents RT-qPCR analysis from a pool of 10 mosquitoes per replicate (3–5 biological replicates). (D) Neutral lipid staining with LD540 (red) shows intense staining in midgut and fat body tissues and weak staining in ovaries from AgTL2-depleted females at 48 hPBM (scale bar = 10 μm for midguts, 50 μm for ovaries and fat body images). Blue = DAPI (DNA). Numerical data supporting this figure is available in the Harvard Dataverse online repository at https://doi.org/10.7910/DVN/ULTW1K. (TIF) [file pbio.3002960.s001.tif]

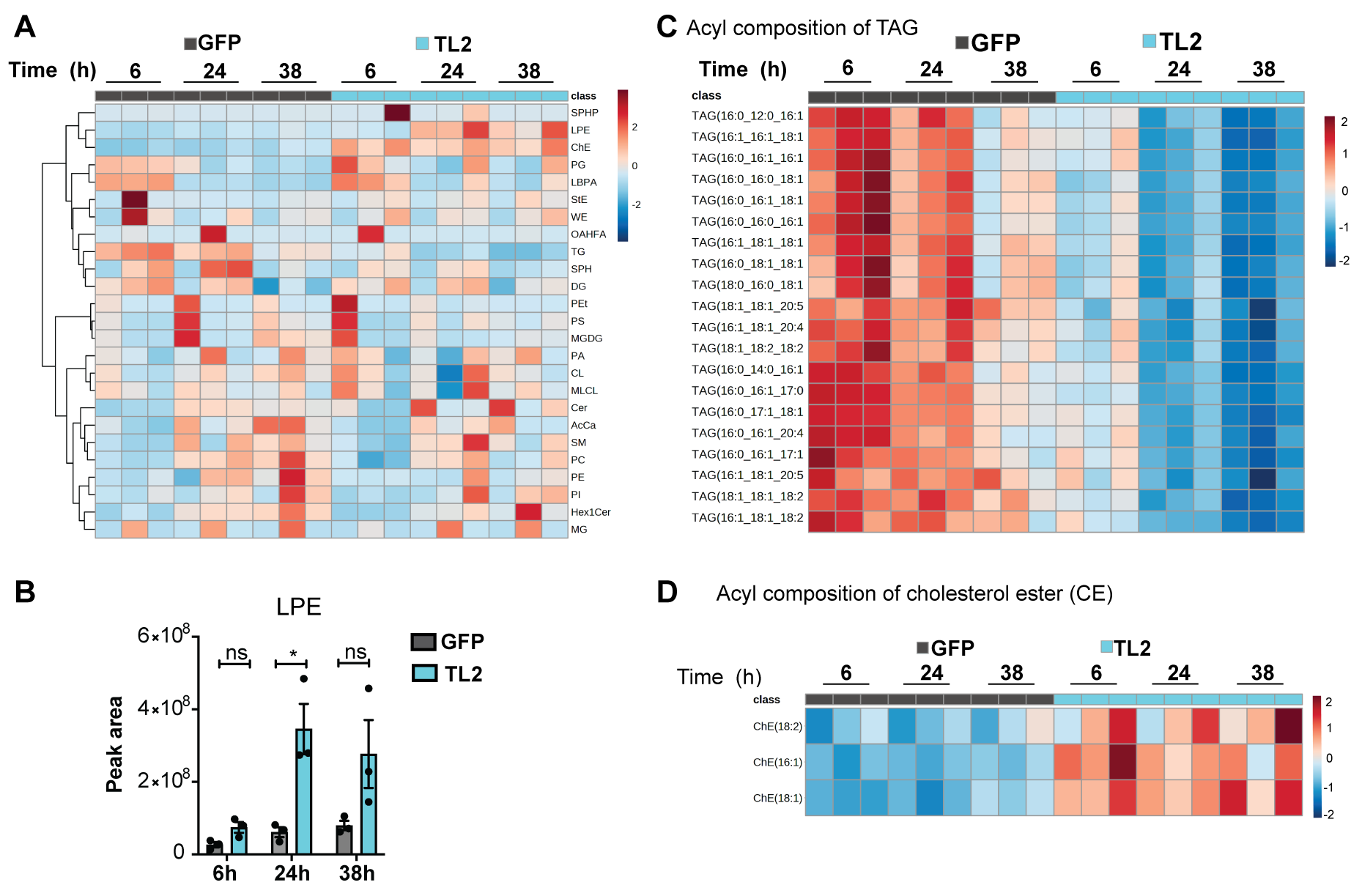

Supplement: S2 Fig — (A) Heatmap of major lipids analyzed by LC-MS reveals dysregulated levels in dsAgTL2 embryos compared to controls throughout development. (B) Lysophosphatidylethanolamine (LPE) levels are significantly increased in dsAgTL2 embryos (Ordinary 2-way ANOVA, Šídák’s multiple comparisons correction, * = p < 0.05). (C, D) Heatmap of acyl composition of (C) top 20 TAGs and (D) 3 cholesterol esters. Heatmaps: values of t test statistic, range: blue to red = significant decrease to increase. Three biological replicates, represented by triplicate columns at each timepoint. See S1 Table and S1 Data. Numerical data supporting this figure is available in the Harvard Dataverse online repository at https://doi.org/10.7910/DVN/ULTW1K. (TIF) [file pbio.3002960.s002.tif]

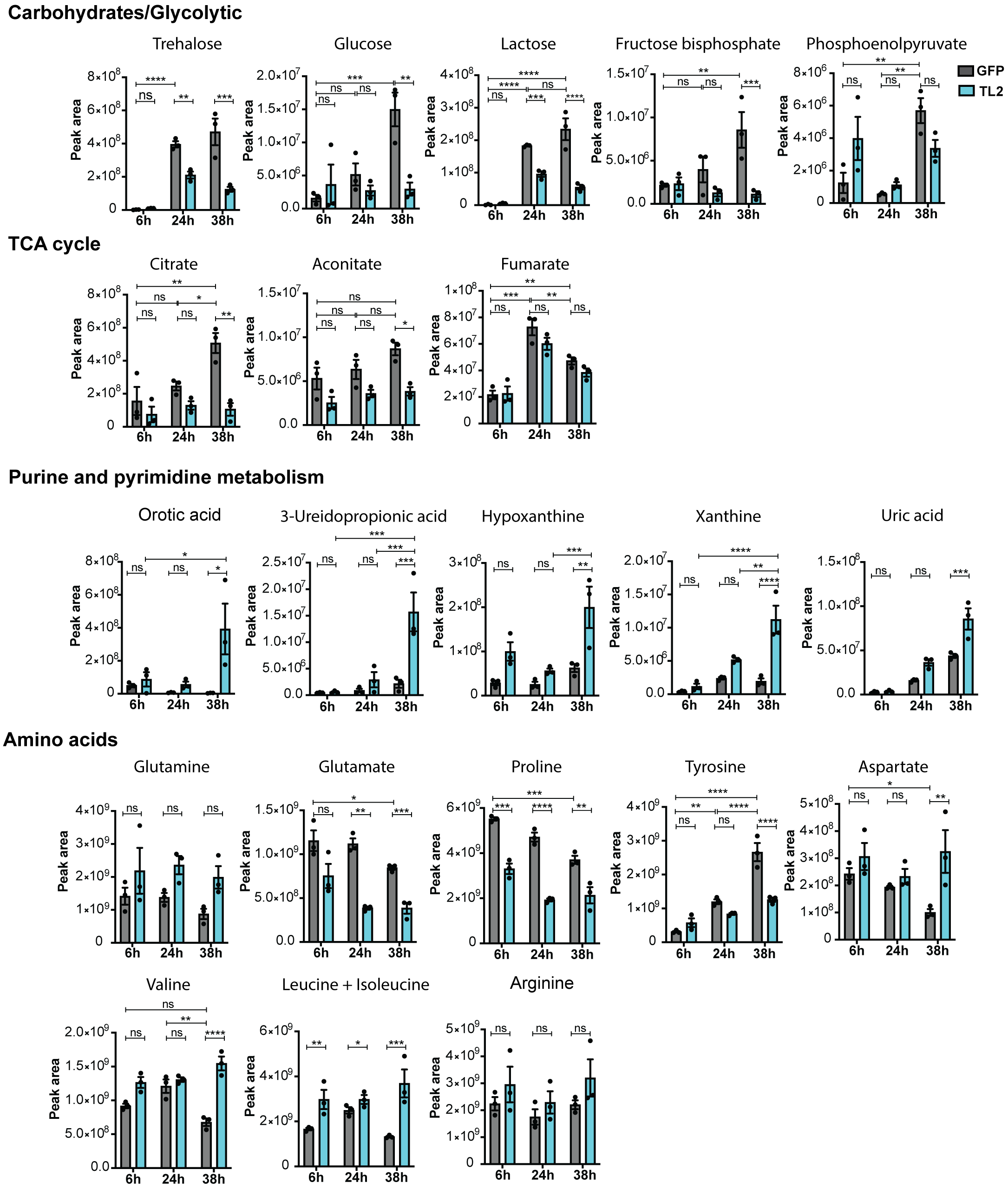

Supplement: S3 Fig — LC-MS analysis reveals dysregulated levels of key metabolites in embryos from AgTL2-deficient females at one or multiple time points after oviposition. Statistical significance was assessed by Least Square Means models testing effect of treatment, time points, and replicate. Adjusted p-values were calculated using an FDR = 0.05. Not all significant comparisons are shown for clarity. See S2 Data. (ns = not significant, * = p < 0.05, ** = p < 0.01, *** = p < 0.001, **** = p < 0.0001) Numerical data supporting this figure is available in the Harvard Dataverse online repository at https://doi.org/10.7910/DVN/ULTW1K. (TIF) [file pbio.3002960.s003.tif]

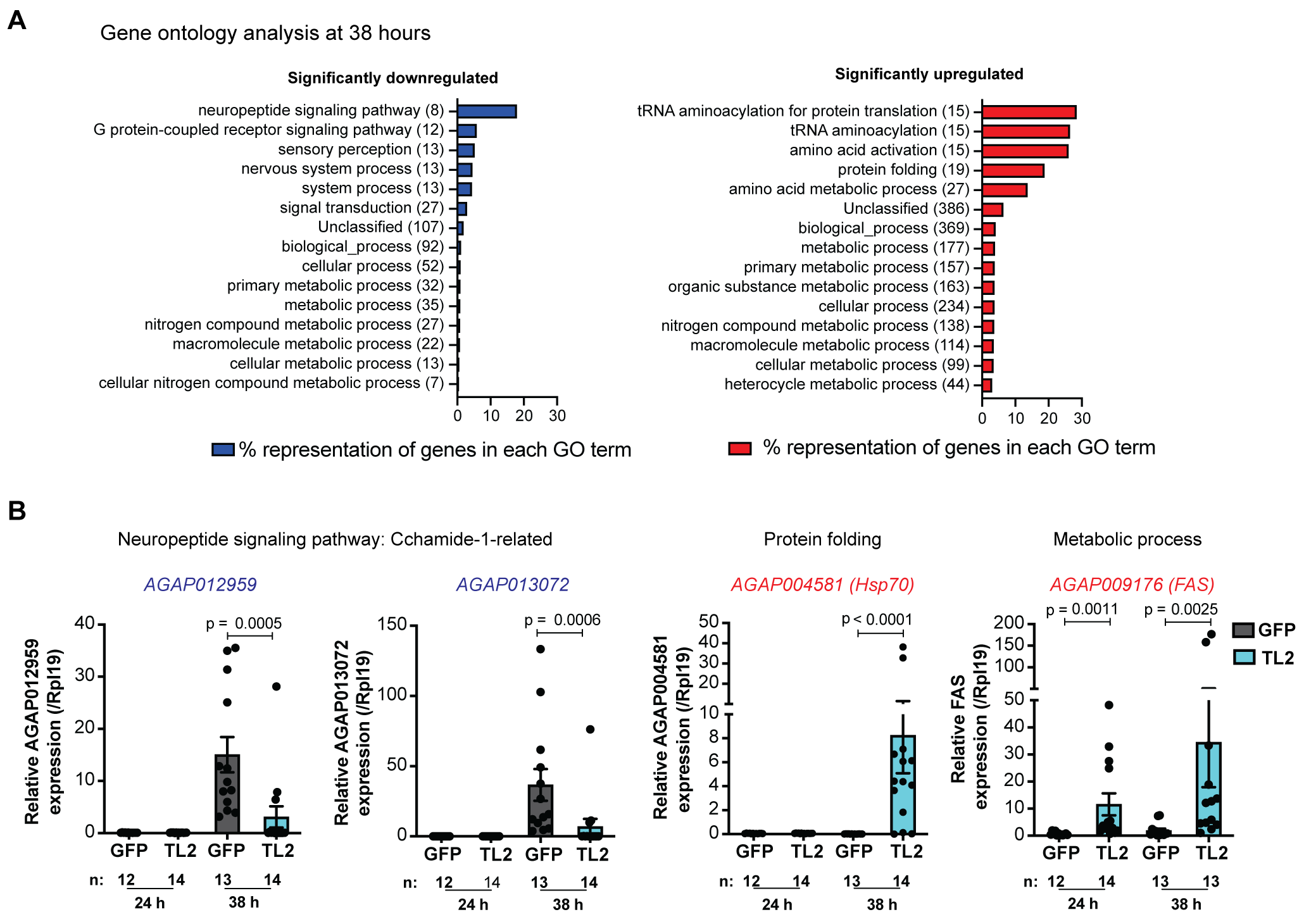

Supplement: S4 Fig — (A) At 38 h post oviposition, genes involved in neuropeptide signaling pathway, sensory perception, and nervous system processes are most significantly represented among down-regulated genes in dsAgTL2 embryos compared to controls, while genes involved in protein translation, protein folding, and metabolic processes are most significantly represented in up-regulated genes (numbers in brackets indicate the number of genes in each data set associated with a biological process). (B) RT-qPCR analyses confirm significantly down-regulation of 2 Cchamide-1-related genes (neuropeptide signaling pathway) at 38 h post oviposition in dsAgTL2 embryos compared to controls, while Heat shock protein 70 (Hsp70) (protein folding) and fatty acid synthase (FAS) (metabolic process) are significantly up-regulated. Kruskal–Wallis test with Dunn’s multiple comparisons correction. See S3 Data. n = number of pools of at least 50 embryos per female from 2 biological replicates. Numerical data supporting this figure is available in the Harvard Dataverse online repository at https://doi.org/10.7910/DVN/ULTW1K. (TIF) [file pbio.3002960.s004.tif]

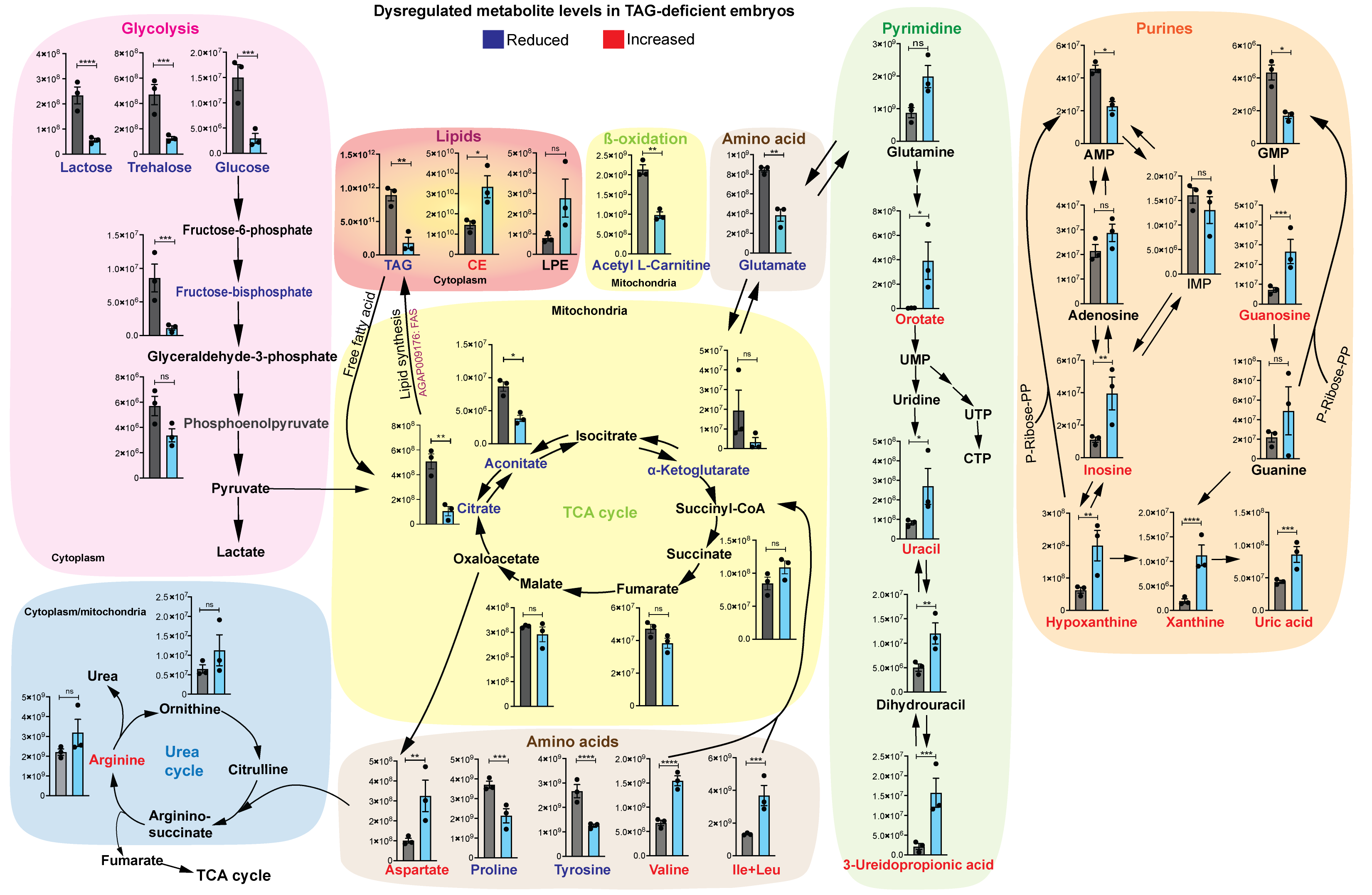

Supplement: S5 Fig — dsAgTL2 embryos have significantly reduced levels of main insect sugars and the glycolytic intermediate fructose-bisphosphate, reduced levels of early TCA and ß-oxidation intermediates citrate, aconitate and Acetyl L-carnitine, altered levels of amino acids, and increased levels of nucleotide degradation products (xanthine, hypoxanthine, uric acid, and 3-ureidopropionic acid). Only data from the 38 h time point are shown (but Least Square Means models testing effect of treatment, time point, and replicate were built on the entire time course). Metabolites highlighted in blue are significantly reduced and those highlighted in red are significantly increased. Adjusted p-values were calculated using an FDR = 0.05. Not all significant comparisons are shown, for clarity. See S3 Fig and S2 Data. (* = p < 0.05, ** = p < 0.01, *** = p < 0.0001, **** = p < 0.0001). Numerical data supporting this figure is available in the Harvard Dataverse online repository at https://doi.org/10.7910/DVN/ULTW1K. (TIF) [file pbio.3002960.s005.tif]

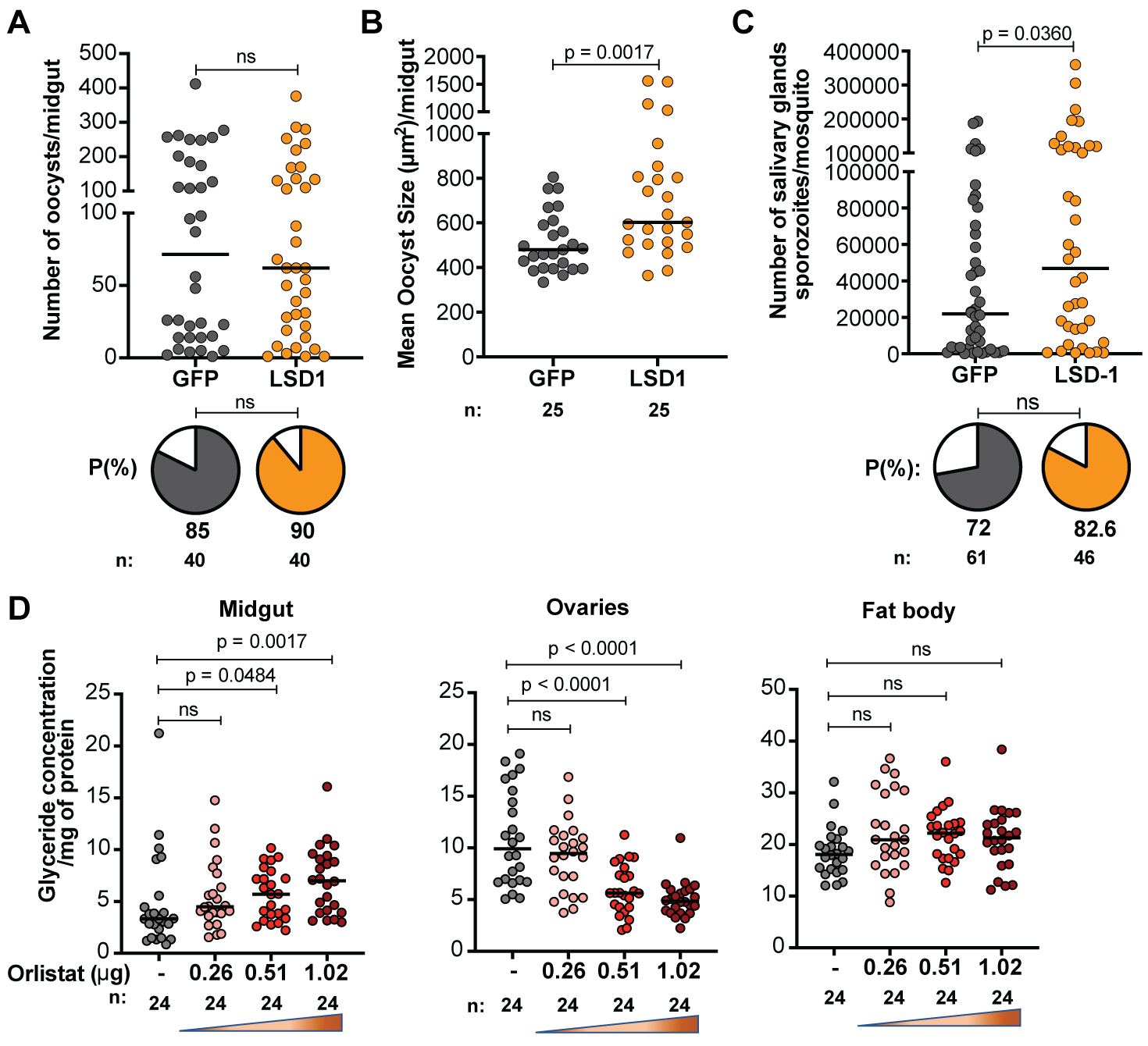

Supplement: S6 Fig — (A–C) AgLSD1 knockdown has (A) no effect on P. falciparum infection intensity (Mann–Whitney) and prevalence (P) (Pie charts, Fisher’s exact, ns; p > 0.05) compared to dsGFP controls but leads to (B) increased oocyst size on 7 d PBM and (C) higher sporozoite numbers in salivary glands at 12–13 d PBM (Unpaired t test). (D) Orlistat treatment leads to increased glyceride levels in midguts and decreased levels in ovaries compared to control-injected mosquitoes, but no changes are observed in the fat body. All data at 48 h PBM (Ordinary 1-way ANOVA, Dunnett’s multiple comparisons correction). Three–four biological replicates are represented in A–C, and 4 in D. n = number of individual mosquitoes analyzed (except for glyceride assay, where n represents the number of pools of 3 insects). Numerical data supporting this figure is available in the Harvard Dataverse online repository at https://doi.org/10.7910/DVN/ULTW1K. (TIF) [file pbio.3002960.s006.tif]
