# Supplementary material for: Maternal lipid mobilization is essential for embryonic development in the malaria vector Anopheles gambiae
Source: PLoS Biol. 2024 Dec 17;22(12):e3002960. doi: 10.1371/journal.pbio.3002960 (PMC11703037; doi:10.1371/journal.pbio.3002960)
Supplement: S1 Table — (DOCX) [file pbio.3002960.s007.docx]

**Table S1 Glossary of abbreviations**

| **ABC** | ATP-binding cassette |
| --- | --- |
| **AcCa** | acyl carnitine |
| **ACTH** | adrenocorticotropic hormone |
| **ADP** | adenosine diphosphate |
| **Ag** | *Anopheles (An.) gambiae* |
| **AKH** | adipokinetic hormone, encoded by two genes AKH1 and AKH2 in *An. gambiae* |
| **AKHR** | adipokinetic hormone receptor |
| **AMT** | Advanced Microscopy Techniques |
| **ANOVA** | analysis of variance |
| **ATGL** | adipose triglyceride lipase |
| **BLAST** | basic local alignment search tool |
| **bmm** | brummer, gene name for ATGL homolog in *Drosophila*, containing a brummer box, a domain sufficient to localize to lipid droplets |
| **BSA** | bovine serum albumin |
| **cAMP** | cyclic adenosine monophosphate |
| **CCHamide** | cyclic neuropeptide containing a disulfide bridge between 2 cysteine amino acids and a C-terminal amidated histidine |
| **cDNA** | complementary deoxyribonucleic acid |
| **CE / ChE** | cholesterol ester |
| **Cer** | ceramide |
| **CerPE** | ceramide phosphoethanolamine |
| **CGI-58** | comparative gene identification-58 |
| **CL** | cardiolipin |
| **DAG / DG** | diacylglyceride/diglyceride |
| **DAPI** | 4',6-diamidino-2-phenylindole |
| **DESeq2** | a script package called “differential expression analysis based on the negative binomial distribution” to find differentially expressed genes using normalized sequencing read counts |
| **dGMP** | 2’-deoxyguanosine 5’-monophosphate |
| **DMSO** | dimethyl sulfoxide |
| **DNA** | deoxyribonucleic acid |
| **DNase** | deoxyribonuclease |
| **dpi** | days post injection |
| **dsRNA** | double-stranded ribonucleic acid |
| **dUTP** | 2’-deoxyuridine 5’-triphosphate |
| **EDTA** | ethylenediaminetetraacetic acid, a divalent cation chelator |
| **eGFP** | enhanced green fluorescent protein |
| **FAS** | fatty acid synthase |
| **FC** | fold change |
| **FDR** | false discovery rate |
| **FFA** | free fatty acid |
| **FMRFamide** | short neuropeptide with the amino acid sequence H-Phe-Met-Arg-Phe-NH2 |
| **Fwd** | forward |
| **GABA** | gamma-aminobutyric acid |
| **GFP** | green fluorescent protein |
| **GMP** | guanosine 5’-monophosphate |
| **GPCR** | G-protein-coupled receptor |
| **Hex1Cer** | hexosylceramide |
| **HHMI** | Howard Hughes Medical Institute |
| **HILIC** | hydrophilic interaction liquid chromatography |
| **HISAT2** | a script called “hierarchical indexing for spliced alignment of transcripts 2” to map sequencing reads to a genome |
| **hPBM** | hours post blood meal |
| **HPLC-MS** | high pressure liquid chromatography followed by mass spectrometry |
| **HRiA** | Health Resources in Action |
| **HSL** | hormone-sensitive lipase |
| **HSP70** | heat shock protein 70 |
| **htseq-count** | a script called “high-throughput sequencing read count” which counts numbers of reads mapped to genes |
| **IDT** | Integrated DNA Technologies |
| **JNK** | c-Jun N-terminal Kinase, a kinase and signaling pathway triggered by cellular stress |
| **LBPA** | lysobisphosphatidic acid, also known as bis(monoacylglycero)phosphate |
| **LC** | liquid chromatography |
| **LC-MS** | liquid chromatography followed by mass spectrometry |
| **LD** | lipid droplet |
| **LD540** | a dye used to stain neutral lipids |
| **Lp** | lipophorin, the major mosquito hemolymph lipid transporter |
| **LPE** | lysophosphatidylethanolamine |
| **LSD1** | lipid storage droplet protein 1 |
| **MB** | maleate buffer |
| **MAG / MG** | monoacylglyceride / monoglyceride |
| **MGDG** | monogalactosyldiacylglycerol |
| **MLCL** | monolysocardiolipin |
| **MPA** | mobile phase A buffer |
| **MPB** | mobile phase B buffer |
| **mRNA** | messenger ribonucleic acid |
| **MS** | mass spectrometry |
| **NAAG** | N-acetyl-1-aspartylglutamic acid |
| **NAD** | Nicotinamide adenine dinucleotide |
| **NBF** | non-blood fed |
| **NF-κB** | Immune signaling pathway mediated by a transcription factor known as “Nuclear factor kappa-light-chain-enhancer of activated B cells“ |
| **NF54** | strain of a laboratory colony of *Plasmodium falciparum* parasites |
| **NGS** | next generation sequencing |
| **NIH** | National Institutes of Health |
| **NP-020454** | NP-020454, an unannotated compound with formula C_6_H_13_NO |
| **NP-40** | Nonidet P-40, a detergent to break open cell and nuclear membranes |
| **ns** | non-significant |
| **NSERC** | Natural Sciences and Engineering Research Council of Canada |
| **OAHFA** | OAcyl-(gamma-hydroxy)FA |
| **PA** | phosphatidic acid |
| **PBM** | post blood meal |
| **PBS** | phosphate-buffered saline |
| **PC** | phosphatidylcholine |
| **PCR** | polymerase chain reaction |
| **PE** | phosphatidylethanolamine |
| **PEEK** | poly(etherether ketone) |
| **PEST** | strain of a laboratory colony of *Anopheles gambiae* mosquitoes |
| **PEt** | phosphatidylethanol |
| **PFA** | paraformaldehyde |
| **PG** | phosphatidylglycerol |
| **PI** | phosphatidylinositol |
| **PKA** | protein kinase A |
| **PS** | phosphatidylserine |
| **qPCR** | quantitative polymerase chain reaction |
| **Rev** | reverse |
| **RNAseq** | high-throughput ribonucleic acid sequencing |
| **RT-qPCR** | real-time quantitative polymerase chain reaction |
| **SM** | sphingomyelin |
| **SPH** | sphingosine |
| **SPHP** | sphingosine phosphate |
| **StE** | stigmasterol ester |
| **SYBR** | nucleic acid dye that binds DNA and fluoresces |
| **TAG / TG** | triacylglyceride/triglyceride |
| **TCA** | tricarboxylic acid |
| **TEM** | transmission electron microscopy |
| **TL2** | triacylglyceride lipase 2 |
| **tRNA** | transfer RNA |
| **Vg** | vitellogenin |
| **WE** | wax ester |
