# Supplementary material for: Maternal lipid mobilization is essential for embryonic development in the malaria vector Anopheles gambiae
Source: PLoS Biol. 2024 Dec 17;22(12):e3002960. doi: 10.1371/journal.pbio.3002960 (PMC11703037; doi:10.1371/journal.pbio.3002960)
Supplement: Attachment — Submitted filename: Response to reviewers.docx [file pbio.3002960.s011.docx]

**Reviewer #1:** 
*This paper presents data on the role of lipids in embryonic development of the malaria vector Anopheles gambiae.  The study reports three main findings: 1) RNAi knockdown of a TAG lipase (AgTL2) increased TAG stores in the midgut and fat body of adult females after blood feeding while reducing them in eggs, 2) knockdown of AgTL2 reduces TAG stores in eggs while knockdown of AgTL2 and a lipid storage droplet protein (AgLSD1) near fully inhibited egg hatching, and 3) a commercially available lipase inhibitor (Orlistat) also reduced egg hatching when applied to surfaces that adult females contact without effect on malaria parasite development.

The manuscript I was sent indicated it was an R1 (revised) submission.  I was not a reviewer of the original submission and was also not given any previous assessments if in fact the paper was previously sent out for review.  With this in mind, my assessment of the manuscript is mixed.  

Strengths include an overall well-written text and associated figures that were easy to read.  Reported results support the main conclusions that inhibition of lipase activity greatly reduces egg hatching.  Results from the Orlistat experiments provide a proof of principle that lipase inhibitors potentially could be used to reduce mosquito fertility.  A third strength from the perspective of mosquito biology is that the oogenesis literature has primarily emphasized the regulation of vitellogenin biosynthesis while focusing somewhat less on the importance of lipid packaging into eggs as a requirement for embryo development.  This last point is something the authors don't actually mention in the manuscript (see below) but is a strength in the eyes of this reviewer.*

We are grateful to the reviewer for these positive comments, and for pointing out that we should emphasize the fact that our work moves the study of oogenesis beyond vitellogenesis. Following these and other comments below, we have added information in the introduction and discussion on the vitellogenesis literature in *Anopheles* and other mosquitoes. We now refer to our recent work showing early embryonic lethality in Vg-depleted *Anopheles gambiae* embryos and the reference literature from the arboviral vector *Aedes aegypti*.

*Per guidelines, short reports in PLoS Biology are supposed to be novel, provocative and of general interest.  I honestly was not persuaded the results were novel/provocative for three reasons:
1.  Background.  The authors begin the paper with a succinct summary of lipolysis in mammals followed by noting that insects possess similar machinery. The authors also introduce adipokinetic hormone (AKH) as a lipolysis activator.  AKH has analogous functions to glucagon in mammals that the authors don't state but should have for general readers.*

Thank you for pointing this out. We have added this information to the revised text, citing Kim and Rulifson 2004 (PMID:15372035), who showed the functional analogy with glucagon in *Drosophila*.

*The papers the authors cite in the Introduction are pretty dated.  They also leave out a fairly substantial literature on the topic across insects generally (many references) and in mosquitoes where a number of recent studies have examined lipid storage, mobilization and packaging into oocytes beginning with Briegel's work in the early 2000s to the present (Pinch et al. 2021 as one of several examples). AKH is a key regulator of lipogenesis in insects but published results in mosquitoes also implicate other hormones in regulating lipid metabolism (see Wang et al. 2017; Dou et al. 2023).*

We apologize for these omissions in references. We have now more comprehensibly and fairly cited the insect literature regarding targeting lipid mobilization pathway components in other insects. This is the text we added to the manuscript:

Dynamic changes occur in the lipid profile of the fat body during a reproductive cycle (27), with substantial proportions of stored lipids being mobilized for egg production (28, 29). Lp-transported lipids, as well as the yolk protein vitellogenin (Vg), are incorporated into developing oocytes (30, 31) whereupon lipid is organized into LDs and Vg crystallizes into vitellin yolk granules (32) to be degraded after oviposition to nourish the developing embryo (33). Coordinated lipolysis regulation is mediated by multiple conserved signaling pathways, including 20-hydroxyecdysone (34, 35), Hedgehog (36), Decapentaplegic (37), NF-κB (38), as well as the neuropeptides FMRFamide (39) and AKH (40). The components of the lipolytic machinery are shared by important insect vectors of human diseases including malaria, Chagas disease and African Trypanosomiasis (23, 41-44), and could potentially be targeted to reduce the size of vector populations and aid in disease control efforts. Orthologues of TAG lipase and AKHR are significantly increased after blood feeding in the fat body of *Aedes aegypti* mosquitoes (34, 40), and TAG stores in the fat body, which are sharply reduced after blood feeding due to their mobilization and incorporation into developing eggs, are not mobilized when the AKH receptor AKHR is silenced by RNAi (40). Similarly, disruption of lipid mobilization through targeting AKH signaling, ATGL or Perilipin/LSD1 in several insects decreases fecundity (43, 45) and causes embryonic lethality (21, 46). However, information regarding lipid mobilization in *Anopheles* is relatively scarce.

*A number of studies in different insects also report results that yolk, which primarily consists of protein and lipid, is essential for embryo development.  This literature goes well beyond Attardo et al. 2012 and Yang et al. 2024 the authors cite in the Discussion.*

We have now discussed the importance of yolk, presenting some relevant literature as also outlined below. As yolk is not the focus of this study, we have only cited some relevant literature on the subject, which has been extensively studied and reviewed in insects.

Besides Lp-transported lipids, the yolk protein Vg conveys ~5% of total oocyte lipids (51), and so yolk deposition could potentially be affected in Ag*TL2* embryos and contribute to their abnormal development. Interestingly, embryonic lethality was reported when maternally-deposited enzymes required for yolk usage in the embryo were silenced in *Rhodnius prolixus* embryos (70), suggesting that such phenotypes are due to an inability to mobilize yolk effectively. Our studies in *An. gambiae* have however shown that embryonic lethality occurs much earlier when *Vg* is silenced. Indeed, Vg depletion in mothers, which led to reduced yolk protein deposition into the oocyte, arrested embryonic development within the first few hours of oviposition (31), suggesting the late mortality observed in dsAg*TL2* embryos is not due to defects in yolk deposition.

*My reason for making these points is two-fold.  First, the Introduction and Discussion leave the impression the lipid storage/mobilization literature in mosquitoes and other insects is thinner than it is.  This may have been unintentional on the part of the authors but also left the impression with this reviewer that the paper addressed an area that is less studied than is actually the case.  I fully recognize A. gambiae as a malaria vector has applied justifications for reporting interventions affecting disease transmission and that reported findings are new for this species.  More fundamentally though, I just didn't see results reporting that macronutrients like lipid are yolk components that embryos require for development as being novel or provocative.*

We agree with the reviewer that there is considerable literature in insects about the role of lipids and lipolysis in embryonic development. However, we respectfully point out that this does not take away from the importance of our study. This study provides a comprehensive analysis of the role of lipid mobilization in a mosquito species of medical importance, painstakingly detailing the events triggered by inhibition of lipolytic enzymes. We demonstrate that lack of remobilization in mothers leads to complete embryonic lethality, and that this lethality occurs very late during embryonic development, preventing embryos from hatching. Additionally, we recapitulate the lethality phenotype using lipase inhibitors, which could be helpful in future control campaigns if mosquito-specific inhibitors were developed. To our knowledge, there is no other study in mosquitoes documenting these events. Although we could not determine the exact mechanisms behind the failed hatching of embryos, we have laid the basis for follow up studies in this area, which could provide novel ideas for mosquito control, for which we provide proof-of-principle experiments using a lipase inhibitor.

*2.  Multiomic data show that a lot of processes change with reduced lipid stores in eggs but these findings are overall hardly surprising given the greater literature in insects, other invertebrates, or vertebrates where externally laid eggs have long been known to depend on packaged nutrients for development.  The multiomic data are thus voluminous but I also came away concluding this information didn't substantially clarify underlying mechanisms for failure of eggs to hatch because so many things go wrong.  A cursory inspection of the mammalian (human) literature makes this emphatically clear where a huge number of defects have been characterized in association with lipid deficiencies.*

*Emphasizing effects on neuronal functions in the Discussion read too much like cherry picking and didn't square with the wider-ranging alterations reported in the results.*

Based on this comment and comments from other reviewers, we have considerably toned down the reference to neuronal deficits and added other possibilities. In the original manuscript we speculated that the lack of hatching may be due to impaired neuronal functions due to the strong neuronal signature observed by RNAseq, but we agree that this remains pure speculation and have now added additional hypotheses, including those suggested by reviewer 3.

*Lastly, I struggled to see how findings are likely to inform studies on embryo development where maternal nutrients are provisioned by other mechanisms that don't involve yolk formation.  Ironically, PLoS Biology published a paper many years ago (Brawand et al. 2008) that looked at how loss of yolk genes in mammals parallel the transition to producing a placenta and other novel machinery (lactation) that provision lipid and other nutrients to embryos.  
As noted above, the mosquito literature has primarily focused on packaging of vitellogenin into eggs, while a novel feature of this study is its focus on the consequences of reducing lipid stores in oocytes.  I would hypothesize a key reason for this is that vitellogenin is actually a precursor for the lipo- and phosphoproteins that are the predominant components of yolk in all animals that lay eggs.  Since the authors make no mention of vitellogenin in the manuscript, I would recommend they consider doing so in the context of reported results.*

As discussed above, we have now added a paragraph on vitellogenin in the introduction and discussion. Interestingly, we published a paper earlier this year (Stryapunina *et al.*, PLoS Pathogens 2024, <https://doi.org/10.1371/journal.pgen.1011145>, PMID: 38285728) that shows that impairing *Vg* expression induces embryonic lethality, but in that case, lethality occurs very early in development (within a few hours of oviposition), compared to the very late lethality observed after AgTL2 silencing. This suggests that the effects of AgTL2 silencing are unrelated to yolk formation. We have now added these results and other relevant Vg literature to the discussion. Of note, previous studies have shown that Vg is converted to vitellin (Raikhel and Lea 1986, PMID: 3750316) and then broken down after fertilization to provide amino acids, rather than being processed into new proteins (Cho *et al.*, 1999, PMID: 10224092).

*3.  Reporting that Orlistat can be topically delivered to A. gambiae and reduce egg hatching is a new result for any insect.  I would be remiss though to not mention the approach near fully follows other studies the authors have published where drugs were added to bednets that reduced parasite loads in A. gambiae.  This again goes to novelty as results follow previous approaches quite closely.  I also noticed the authors used Orlistat at a dose of 1 mmol/m2 to reduce fertility which at retail costs works out to be about $20/m2 which is massively higher than any currently used insecticide.*

The use of Orlistat provides a proof of principle that lipase inhibitors can induce complete embryonic lethality in *Anopheles*, which had never been shown before. We have now further clarified in the results and discussion that Orlistat is obviously not a suitable compound for mosquito control but that there would be the need to identify mosquito-specific inhibitors for this purpose, if feasible. Therefore, the question of costs of the compounds is not applicable to this study at this stage. We have now also highlighted the challenge of finding specific inhibitors given the similarities of lipolytic machineries in mosquitoes and other organisms, including mammals.

*Points that should be revised if the paper moves forward in PLoS Biology or another journal.
1.  Fig. 1B.  Unclear from the methods or figure legend what is being shown.  Are these oviposited eggs and the Y axis is showing number of eggs laid per GFP or TL2 treated female?  If yes, how was it discerned the eggs developed versus the eggs were simply laid?  Same questions apply to Fig. 1F, Fig. 4B.  Thus, please provide clarification for what egg development refers to.*

In Fig. 1B, 1F and 4B we show the total number of eggs that were laid. No eggs were retained. Therefore, the number of eggs laid is the same as the number of eggs developed. We have now changed the Y-axis to better describe the data and clarified this point in the figure legend and methods.

*2.  Orlistat treatment.  The authors should state in the methods whether Orlistat was dissolved in DMSO and that 207 nl of Orlistat in pure DMSO was injected into females.  I raise this point because many insects respond extremely poorly to injection of even small amounts of DMSO into the hemocoel.*

We have now clarified this protocol in the methods. Briefly, 5.0–6.7 mg orlistat was dissolved in 50 µl pure DMSO to give a 200–270 mM stock solution. It was then diluted as required with 1X PBS to 10 mM working solution, resulting in 3.7–5.0% DMSO in PBS. The 5 mM and 2.5 mM orlistat solutions were prepared by two-fold serial dilution, resulting in 1.85–2.5%, and 0.925%–1.25% DMSO in 1X PBS. The control DMSO group was injected with the same DMSO concentration as in the 10 mM orlistat group (i.e. 3.7–5.0%). The equivalent volume in 207 nl is therefore maximally ~10 nl of DMSO in the mosquito hemolymph (~2 µl), or a final concentration of ~0.5%, which we believe to be negligible. Again, comparisons are made to our controls receiving the same or lower concentrations of DMSO in 1X PBS.

*3.  This study did not examine the underlying mechanisms regulating lipolysis in A. gambiae which in the mosquito literature has primarily been studied in A. aegypti.  This is fully fine given short report guidelines for PLoS Biology.  However, the older literature also provides hints oogenesis may not be fully conserved between culicine and anopheline mosquitoes.  Given egg formation is activated by blood which contains lipid as well, it may be more significant than noted that TAG levels in the midgut were more strongly affected by TL2 knockdown than in the fat body (Fig. 1C, D).  Along these lines, Fig. S1 reports AgTL2 knockdown efficacy in the fat body and 'rest of body' but not in the gut or ovaries, which could also have roles in reducing TAG stores available to embryos.*

It is true that there are reported differences in lipid mobilization between culicine and anopheline mosquitoes. We also wondered if despite being a gene predominantly induced in the fat body after blood feeding, AgTL2 has functions in other tissues and if knockdown was efficient elsewhere. We analyzed AgTL2 expression in both the midgut and ovaries. AgTL2 was detectable in both organs, but levels were not significantly altered by blood feeding (see provided data). Though trending to decrease, we did not detect a significant change in TL2 expression in either the midgut or ovary at either 24 or 48 h pBM following dsTL2 knockdown, leading us to conclude our phenotype is driven by AgTL2 function in the fat body, where knockdown was much more efficient. We will now include these data in the manuscript in panels A and B of S1 Fig (shown below).

**S1 Fig (updated panels A and B)**. (A) Ag*TL2* is significantly induced in the fat body but not the ovaries or midgut of females at two time points after blood feeding (hPBM = hours post blood meal) compared to non-blood fed (NBF) controls. (Ordinary 2-way ANOVA, Šídák’s multiple comparisons correction) (B) Ag*TL2* levels after blood feeding are significantly reduced upon RNAi injections in the fat body but not the ovaries or midgut (Ordinary 2-way ANOVA, Šídák’s multiple comparisons correction).

*It also would be valuable to know if knockdown of the genes the authors targeted actually alter yolk deposition into oocytes which is not apparent from reporting lower TAG levels in whole ovaries, egg laying data, or the morphological data presented in Fig. 2 which only shows late stage (post-gastrulation) embryos.  All this to say the authors are assuming phenotypes are due to suppressed lipolysis in the fat body which may not fully be the case given the results presented.*

As discussed above, we showed earlier this year (Stryapunina *et al.*, PLoS Pathogens 2024, <https://doi.org/10.1371/journal.pgen.1011145>, PMID: 38285728) that impairing *Vg* expression induces embryonic lethality much earlier in development, which excludes yolk deposition as a cause of the late lethality observed here. We have now added mention of these results and other relevant Vg literature to the discussion. We also have a western blot showing that Vg is deposited and vitellin processed normally in AgTL2 embryos. We do not think we should add this information to the manuscript, but we attach it here as this reviewer may find it interesting.

**Additional Data Figure 1: Vg levels in embryos are not decreased by AgTL2 silencing.** Actin and Vg protein levels were detected using specific antibodies at indicated timepoints after oviposition of 200 embryos from ds*GFP* and ds*TL2*-injected mothers. As expected, actin levels increased during embryogenesis, whereas Vg as vitellin was degraded from a ~240 kDa protein (top band clearly visible in 4–6 h AgTL2 embryos) into smaller protein fragments.

**Reviewer #2:** 
*This is a 'short report' by Itoe and coworkers on lipid mobilization in female Anopheles gambiae. They knocked down two genes - TL2 and LSD1 - a lipase and a Lipid storage droplet protein and observed strong distinct phenotypes in egg and embryo development and Plasmodium development using lipidomics, metabolomics, and RNAseq. This is a really nice study and the manuscript is in very good shape. I have really only some minor comments.*

We thank this reviewer for their supportive comments.

*Line 91/148 'impared neuronal function' is pure speculation. Show evidence or take it out...*

We agree with the reviewer that this was a speculative comment and have now removed this from the abstract, and considerably toned it down in the rest of the manuscript, where we have added other possible explanations.

*Line 173 to 179 I understand the motivation of the authors to present lipase inhibitors as potential future insect management tools. However, there is virtually no chance that this will work out. Mosquito control is about killing mosquitoes and not sterilizing them, with the exception of sterile insect technique. I recommend to remove this language.*

We respectfully disagree with this point. There are dual bed nets, currently utilized in malaria endemic regions, that, besides an insecticide, possess a second compound that induces sterility in mosquitoes to limit the spread of insecticide resistant traits to the surviving progeny. These dual compound nets contain a growth regulator (pyriproxyfen) that reduces fertility (for instance, check:
Grisales *et al.*, 2022 - <https://malariajournal.biomedcentral.com/articles/10.1186/s12936-021-03794-z>;
Tiono *et al.*, 2018 - <https://www.thelancet.com/journals/lancet/article/PIIS0140-6736(18)31711-2/abstract>;
Accrombessi *et al.*, 2023 - <https://www.thelancet.com/journals/lancet/article/PIIS0140-6736(22)02319-4/fulltext>;

Mosha *et al.*, 2022 - <https://www.thelancet.com/journals/lancet/article/PIIS0140-6736(21)02499-5/fulltext>)

Therefore, the idea of using sterilizing compounds is scientifically sound and generally accepted in the malaria control field. For these reasons we have decided to keep this sentence, but have expanded the discussion of the use of sterilizing compounds in dual nets and have highlighted the challenges of generating specific lipase inhibitors for mosquitoes.

*Line 423 Did the experimenters really use field collected mosquitoes for their experiments? That is hard to believe... Please give a more detailed description of their origin. How could anyone repeat these experiments when no established laboratory strain was used?*

We are sorry for any confusion. The *Anopheles gambiae* G3 mosquitoes utilized in this study are the standard *Anopheles gambiae* colony that is maintained and studied in several malaria laboratories worldwide. We have now clarified in the methods that these are a laboratory colony and not field mosquitoes: *“Anopheles gambiae* (the standard laboratory wild-type strain G3)”.

*Line 549 How do you determine fertility with a stereo microscope? It is impossible to distinguish dead and living eggs. Please explain.*

We have now changed the language to clarify that the hatching of the embryos was determined with the help of a microscope: “One week after oviposition, the number of eggs laid, and the fertility was determined using a Nikon SMZ100 dissecting scope. Specifically, fertility was assessed by the degree of melanization of eggs laid (black for fertile and brown for infertile), followed by needle pricking of unhatched eggs to assess the presence (or absence) of fully developed larvae”.

*Supplemental Figure 1 C. Check labels.*

Thank you for catching this error. We have corrected the graph label to LSD1.

----------------------------------------------------------------

**Reviewer #3:** 
*The manuscript "Maternal lipid mobilization is essential for embryonic development in a key malaria vector" presents significant findings on the role of lipid mobilization in the reproductive biology of Anopheles gambiae. The research demonstrates that impairing lipolysis in female mosquitoes leads to embryonic lethality, providing insights into potential new strategies for mosquito control. The paper is well written and the study is well-conducted, the data are robust and the figures are clear and easy to interpret.*

We are grateful to this reviewer for their supportive comments

*However, there are a few areas where the manuscript could be strengthened in terms of interpretation of the embryonic neuronal phenotype and potential challenges associated with implementing a mosquito control solution based on inhibition of lipid metabolism. Some conclusions, such as attributing all developmental issues to impaired neuronal functions, may be overinterpretations. It would be good to highlight that potentially multiple factors could be involved and suggest areas for further research.*

This point was also raised by other reviewers. We agree the link to neuronal deficits is speculative and have now removed it from the abstract and toned it down in other parts of the manuscript, while adding possible alternatives.

*The discussion on how lipid deficiencies affect neuronal development and function should be expanded to include possibilities beyond energy metabolism as the reason for nervous system disruption. Mention the various roles of triglycerides and their derivatives in the nervous system, such as membrane synthesis/synapse function, production of key signaling molecules from fatty acids and cholesterols, and myelination.*

We thank the reviewer for these suggestions, which have been incorporated into the manuscript.

*I think something else that would be useful is consideration of the potential issues and difficulties that would arise from targeting lipid metabolism given how conserved these systems are across biology. It is important to discuss the potential ecological impact of targeting lipolysis, particularly concerning non-target organisms in addition to the safety concerns of using lipase inhibitors in mosquito control, especially regarding potential effects on humans who handle and sleep in close contact with these bednets. Given the conserved nature of lipolysis, this is a significant concern that needs to be discussed. A more detailed examination of this risk would enhance the paper's credibility by demonstrating consideration of broader ecological consequences. It is mentioned to generate an Anopheles specific inhibitor, but it would be good to expand upon the research required and challenges associated with the production of such an intervention.*

We completely agree with this comment and have now significantly changed the discussion of the possible application of lipolysis inhibitors for malaria control. We have expanded on the issue of the conserved nature of this process, and have highlighted the risks for non-target organisms. The section is below:

Orlistat is a drug used in humans to counter obesity and therefore it would not be suitable for a mosquito-targeting strategy. However, in the future, it may be feasible to generate *Anopheles*-specific inhibitors that target the function of AgTL2, AgLSD1 or other factors critical to mosquito lipolysis, for incorporation in bed nets (or other vector control strategies like indoor residual sprays (64)). Developing these inhibitors for mosquito control may prove challenging given the close homology between mosquito and human lipases (21), although it is possible that allosteric inhibitors may show higher specificity against mosquito enzymes (65). Potential impacts on nontarget organisms would need to be evaluated through toxicological testing in mammals and insect surveys during efficacy trials of the intervention. If necessary, effects could mitigated through usage methods, dosages or formulations that reduce exposure of other insects to these inhibitors, such as physical incorporation into bed nets, which generally only target night-biting species contacting the net.

*Also, make sure all the abbreviations are defined at first use and consider adding a glossary of abbreviations at the beginning of the manuscript for the benefit of the reader.*

We have provided a glossary of all abbreviations used in the manuscript as a supplementary table.

*With these revisions, I believe the manuscript will make a substantial contribution to the field. The findings are significant and could potentially inform new strategies for controlling malaria vectors. I recommend acceptance with minor revisions.*

Thank you for these comments, we are glad that you found our findings significant.
